# The Army Medical Corps

**Published:** 1906-11

**Authors:** 


A Monthly Review of Medicine and Surgery.

*EDITOR:*

WILLIAM WARREN POTTER, M. D.

All communications, whether of a literary or business nature, books for review and exchanges, should be addressed to the editor 284 FRANKLIN ST., BUFFALO, N. Y.

VOL. LXII. NOVEMBER, 1906. NO. 4

### The Army Medical Corps.

THERE is every prospect that the long delayed and much needed expansion of the medical corps of the United States Army will be brought about early in December at the coming session of Congress. The delays which have beset the efforts of the Surgeon General of the army to secure legislation bringing about the reforms he has planned, have been so persistent that a less sincere man would have been discouraged; he would have felt the pressure of officialdom, that dank, smothering, enveloping atmosphere which has strangled so many progressive movements in so many directions from almost time immemorial. It may not always have been because the reforms suggested were not recognised as necessary; possibly in some cases it has been because it was "inadvisable at this time;" yet in this particular instance, the expansion of the medical department of the army, the delays have worked a grave danger and placed the members of the department in an unenviable position, to say the least. Here is a corps of men, highly educated in technical medicine, bound down by tradition, kept far below a numerically efficient force by legislative unconcern, which spends lavish millions at large and squeezes the dimes which are given to one of the most important departments of army administration.

The army medical department has not been allowed to keep pace with army expansion, and the result has been that the numerical strength of the medical corps has been far below an efficient figure. To make up the deficiency, which was impossible of addition by reason of legislative restrictions, it was, and has been for years, necessary to employ civilian physicians. Up to a short time ago these physicians were known as "acting assistant surgeons" and were given relative rank of first lieutenants. They were permitted to wear the uniform of a United States army medical officer, with the exception that the collar insignia, and the shoulder straps were of silver instead of gold. That was the line of separation, the identification mark, between the commissioned medical officer and the acting assistant surgeon.

Even at that, with the benefits and privileges which he was permitted to enjoy, the acting assistant surgeon was giving more to his country than any other individual who served in the army, and for this reason: In the event of illness, disability or death, his usefulness to his country was at an end; his contract was annulled and he was dropped. He was not eligible to apply for a pension; in the event of his death his family, however dependent they might be, would have no official right to Government aid. If he lived and were to return to civil life without injury, he had done his duty and was merely fortunate. He had performed professional services which in civil life would have paid him many times the $150 per month which his contract brought him; yet in all the service which acting assistant surgeons have put in there has yet to be heard a single complaint of the amount of money paid for the services rendered. That, apparently, has always been a secondary consideration. The real complaint has been one of lack of fairness, and surgeons general for years past have made futile efforts to correct the evil. In spite of this, in spite of the protests of the profession, the civilian physician was further humiliated by being deprived of even the title of "acting assistant surgeon" and was officially made a "contract surgeon" with the right to wear the letters "C. S." on the collar of his "uniform." As a "contract surgeon" his official position was a joke. He in many cases was the only medical man attached to a command; the health and physical well-being of the fighting force was in his keeping and, although he was officially entitled to be "shown respect" by the enlisted man, he was an official cipher in spite of the fact that he was performing all the duties of a commissioned officer with the exception of sitting on courts-martial.

The one place in officialdom where he was regarded as more than a mere civilian attached as a sort of a necessary barnacle to the army, was the Surgeon General's office, and General O'Reilly and his staff have done their utmost to place him in a more dignified position. Army surgeons received him and treated him as their equal always.

A bill was prepared two years ago increasing the strength of the medical corps and providing for the formation of a reserve corps, and its passage will remove all the obstacles which now beset the surgeon general's office in the proper and efficient administration of the medical department. The bill was approved by the secretary of war and the general staff; President Roosevelt gave it his hearty approval and it was introduced in Congress. It passed the senate and was sent to the house committee, which reported it favorably after slight amendment. There was another army bill under consideration at the time, a bill affecting the Ordnance department. The Speaker did not, for some occult reason, see the wisdom of permitting both bills to go through during the same session and naturally the medical bill was held up, but with a positive promise that it would become a law in December of this year.

All that is necessary now is to have the bill called up. It will then be passed by the house and the conference and final passage will be a matter of form. The medical department of the army will then be placed on a numerically efficient basis and the medical reserve corps will be the nucleus of a body of well-trained medical men who will be available for service in case of necessity. And they will have rank; for with their appointment they will be commissioned as first lieutenants and assistant surgeons of the reserve corps. They can never attain any higher rank in the corps; they will be paid only when actually on duty with troops, but they will be ready for service; they will be conversant with the multiplicity of executive detail which falls to the lot of the army medical officer; they will understand the "paper work" of the army; they will know how to care for government property and there will be a compact, well-trained body of efficient medical officers at the command of the surgeon general, who may be called on for efficient service should necessity arise.

For years the army has been notoriously deficient in medical officers, and never was the need for an increased corps more apparent than at the present time, with the large body of troops on duty in the Philippines and quite 7,000 doing service in Cuba.

It has been a penny wise, pound foolish attitude which has delayed the increase of the corps up to this time and now that relief is so close at hand, there should be concerted effort on the part of the profession to aid the calling up of the bill which is on the eve of passage. And every physician can aid in this by requesting the earnest co-operation of the congressman of his district.

